# P-899. Strong Hearts Program: Results of a Novel Primary-Care Based Chagas Disease Care Program in East Boston, MA, 2017-2023

**DOI:** 10.1093/ofid/ofae631.1090

**Published:** 2025-01-29

**Authors:** Javier J Huerta, Ashley Maldonado, Carly Milliren, Jillian Hertig, Katherine Collins, Jim Gomes, Alejandra Salazar, Daniel Bourque, Natasha Hochberg, Davidson Hamer, Leonard L Levin, Jaime Gallegos Salazar, Elizabeth Barnett, Juan Huanuco, Jennifer Manne-Goehler, Julia Köhler

**Affiliations:** OHSU-PSU School of Public Health, Portland, Oregon; Boston Children's Hospital, Boston, Massachusetts; Boston Children's Hospital, Boston, Massachusetts; Medford Department of Public Health, Medford, Massachusetts; Strong Hearts Chagas Disease Screening Project, Boston, MA; Boston Children's Hospital, Boston, Massachusetts; Boston Medical Center, Boston, MA; Boston Medical Center / Boston University School of Medicine, Boston, Massachusetts; Boston University School of Medicine; Section of Infectious Diseases, Boston University Chobanian & Avedisian School of Medicine, Boston, Massachusetts; Harvard Medical School, Boston, Massachusetts; East Boston Neighborhood Health Center, Boston, MA; Boston Medical Center, Boston, MA; East Boston Neighborhood Health Center, Boston, MA; Division of Infectious Disease, Brigham and Women's Hospital and Harvard Medical School, Boston, Massachusetts; Boston Children's Hospital, Boston, Massachusetts

## Abstract

**Background:**

Chagas disease is a neglected parasitic infection that affects ∼300,000 people in the US, with a prevalence of ∼49 per 100,000 in Massachusetts, USA. Untreated Chagas disease leads to irreversible cardiac morbidity and death in 20-30% of cases, yet < 1% receive antitrypanosomal therapy in the US. We describe Chagas disease epidemiology and the care continuum of the Strong Hearts Program, an initiative centered at the East Boston Neighborhood Health Center (EBNHC) in Boston, MA.

**Fig. 1.**
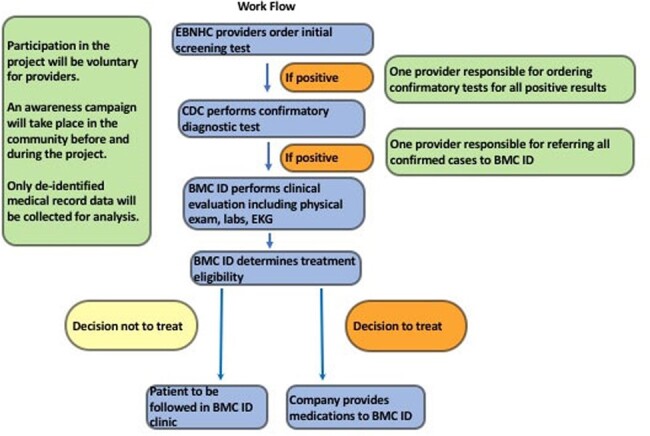
Workflow for Chagas disease care at East Boston Neighborhood Health Center

Clinicians receive support from Strong Hearts volunteers and/or care navigators for the confirmatory process at CDC and for ensuring completion of the specialty clinic referral process.

**Methods:**

Extracted from EBNHC electronic medical records, diagnostic uptake and prevalence were analyzed, stratified by key demographic characteristics. We used chi-squared tests to compare differences in proportions across groups. We abstracted information from the medical records of Chagas patients to identify barriers in the continuum of care, addressed and documented by Strong Hearts’ patient care navigators.

**Fig. 2.**
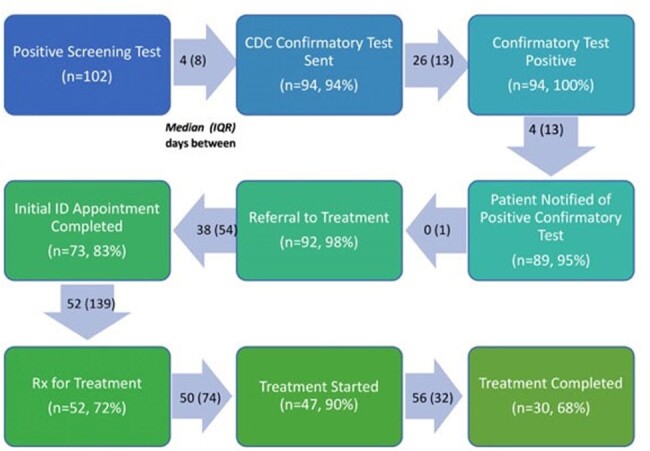
Continuum of care for Chagas disease as supported by the Strong Hearts project. Abbreviated overview of the Chagas disease care continuum of a subset of 90 EBNHC patients diagnosed between 3/2017 - 9/2022. N and % of each step (rectangle) refer to patients having completed the previous step as denominator. Elapsed time between steps is given as median days (on arrows between steps), with IQR in parenthesis.

**Results:**

Following or concomitant with 10 provider- and > 20 community information sessions in East Boston, 14,354 patients were screened at EBNHC 3/2017 - 5/2023, using a protocol approved by the EBNHC board (Fig. 1). Confirmed Chagas patients were referred to Boston Medical Center (BMC) for further evaluation and treatment if indicated. Per quarter, a median of 572 patients were screened at EBNHC (IQR: 393 – 712). The Chagas prevalence in the population was 0.7% (95% CI: 0.6% – 0.9%) with no sex difference. A significant age gradient showed the lowest prevalence in < 20 year olds (0%) and the highest in 40-49 year olds (0.8%; p=< 0.001). Of a subset of 90 EBNHC patients, 44 (49%) began and 28 (31%) completed antitrypanosomal therapy in the BMC Infectious Disease clinic (Fig. 2). Major barriers to diagnosis and treatment included complexity and delays of confirmatory testing at CDC and barriers within the medical system, the latter rectifiable if prioritized by administrators and clinicians. Outside barriers included patients’ inability to take time off from work, obtain child care or transportation.

**Conclusion:**

Given the significant prevalence of Chagas disease in the US, increased patient access to diagnostics, therapy and cardiology follow-up are needed. We find that Chagas care by motivated primary care providers is feasible with appropriate support.

**Disclosures:**

**Daniel Bourque, MD**, Kephera Diagnostics: Grant/Research Support **Natasha Hochberg, MD, MPH**, Novartis: Employee|Novartis: Stocks/Bonds (Public Company) **Davidson Hamer, MD**, CDC, Parasitic Diseases: Grant/Research Support|Kephera: Grant/Research Support

